# Rapid isolation of rare targets from large fluid volumes

**DOI:** 10.1038/s41598-020-69315-1

**Published:** 2020-07-27

**Authors:** Per Niklas Hedde, Margaux Bouzin, Timothy J. Abram, Xiaoming Chen, Melody N. Toosky, Tam Vu, Yiyan Li, Weian Zhao, Enrico Gratton

**Affiliations:** 1 Department of Biomedical Engineering, University of California, Irvine, CA USA; 2 Department of Biochemistry, University of Hawaii at Manoa, Manoa, HI USA; 3 Physics Department, Università degli Studi di Milano-Bicocca, Milan, Italy; 4 Velox Biosystems, Irvine, CA USA; 5 Department of Pharmaceutical Sciences, University of California, Irvine, Irvine, CA USA; 6 Department of Physics and Engineering, Fort Lewis College, Durango, CO USA; 7 Sue and Bill Gross Stem Cell Research Center, University of California, Irvine, Irvine, CA USA; 8 Chao Family Comprehensive Cancer Center, University of California, Irvine, Irvine, CA USA; 9 Edwards Life Sciences Center for Advanced Cardiovascular Technology, University of California, Irvine, Irvine, CA USA; 10 Department of Biological Chemistry, University of California, Irvine, Irvine, CA USA

**Keywords:** Biotechnology, Engineering

## Abstract

Rapidly isolating rare targets from larger, clinically relevant fluid volumes remains an unresolved problem in biomedicine and diagnosis. Here, we describe how 3D particle sorting can enrich targets at ultralow concentrations over 100-fold within minutes not possible with conventional approaches. Current clinical devices based on biochemical extraction and microfluidic solutions typically require high concentrations and/or can only process sub-milliliter volumes in time. In a proof-of-concept application, we isolated bacteria from whole blood as demanded for rapid sepsis diagnosis where minimal numbers of bacteria need to be found in a 1–10 mL blood sample. After sample encapsulation in droplets and target enrichment with the 3D particle sorter within a few minutes, downstream analyses were able to identify bacteria and test for antibiotic susceptibility, information which is critical for successful treatment of bloodstream infections.

## Introduction

**Figure 1:**
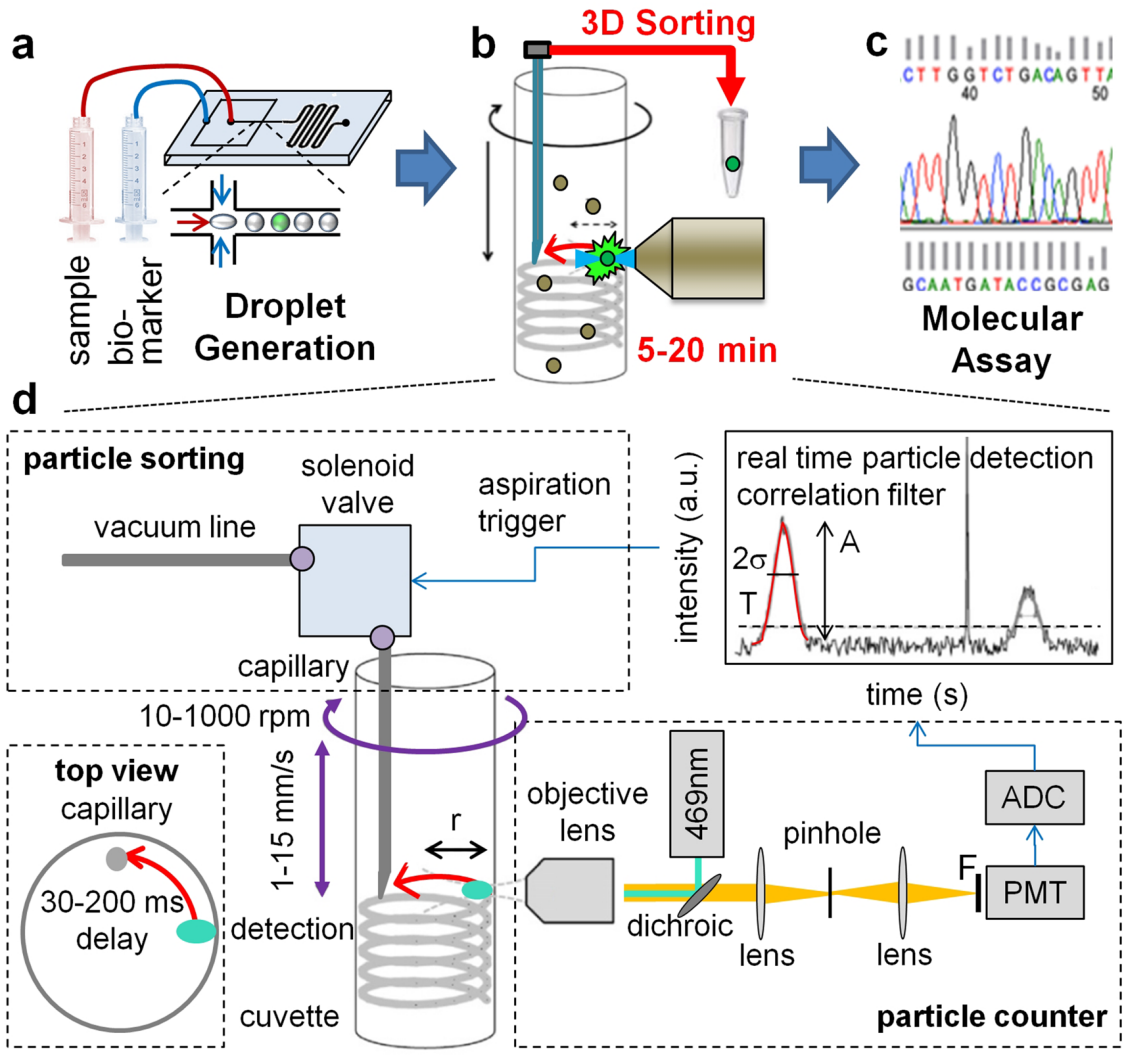
3D particle sorting method and setup. (**a**–**c**) Workflow of 3D particle sorting for the isolation of rare targets from multi-mL clinical samples such as blood. (**a**) Instead of lengthy sample enrichment, for example by blood culture, we encapsulate the sample with fluorescent biomarkers into microdroplets to activate fluorescence in positive droplets. (**b**) These can then be detected and sorted out within minutes using the 3D particle sorter. (**c**) Finally, enriched, sorted aliquots can be subjected to downstream analysis such as sequencing to characterize targets. (**d**) 3D Particle Sorting Method and Setup. The sample was placed into a cylindrical glass cuvette that was rotated (~ 200 rpm) and moved up and down (~ 1 mm/s) in front of an objective lens (NA 0.4) to rapidly scan (~ 0.5 mL/min) a large sample volume (1–8 mL). The detected signal was analyzed in real time with a correlation filter-based algorithm. Upon detection of a positive event, a trigger signal was sent at an appropriate time delay (70 ms) to briefly (5–20 ms) open a solenoid valve connected to a vacuum line to extract a small volume containing the positive particle into a needle or capillary. After extraction of the desired number of particles, the contents can be dispensed into appropriate containers for downstream analyses.

A fundamental issue towards advancing medical diagnostics and therapeutics development is the rare targets or “needle-in-a-haystack” problem, which is still unresolved today^1^. The timely detection of a few bacteria, viruses, cancer cells, specific antibody-producing immune cells, HIV reservoir cells, and other particles in milliliters of bodily fluids such as blood remains a major challenge^2–5^. Target rarity is usually exacerbated by the presence of vast amounts of other bioparticles that prohibit biochemical isolation. Importantly, the detection of a few bacteria in milliliters of blood required for diagnosis of blood stream infections (BSIs) resulting in sepsis typically requires bacteria enrichment by blood culture. Blood culture enrichment can take 24–72 h followed by bacterial identification (ID) and antibiotic susceptibility testing (AST), typically within an additional 4–24 h. Yet, treatment initiation with suitable antibiotics within the first hours of illness can determine life or death^6^. To generate contrast between rare targets and background, fluorescence labeling in combination with optical detection can be employed. Specific bioparticles such as bacteria, viruses, or immune cells can be highlighted by encapsulating the sample fluid into tens of millions of picoliter-sized microdroplets together with fluorescent biomarkers that light up positive droplets^7^. Target labeling, however, demands a choice of strategies often unable to unify sensitivity and specificity. A less specific label such as a metabolic dye can be employed to detect the general presence of a certain group of particles, for example, bacteria in blood. Yet, with nonspecific labeling, important target parameters such as bacterial subspecies and antibiotic susceptibility profiles remain unknown, a lack of specificity which prohibits efficient treatment. If, on the other hand, a label tailored to a certain subtype of particles is employed, for example, a piece of DNA sequence matching a specific strain of bacteria^7^, other variations of the same particle family will remain undetected. In a clinical setting with many possible unknown targets, the large number of repeated specific tests required to achieve high test sensitivity is often not compatible with limited sample amounts and a demanding time-to-result. To break this contradiction, testing can be separated into two steps. First, less specific labeling identifies particle candidates that are then isolated to enable highly specific tests, such as single cell sequencing, in a second step. By droplet encapsulation, sample components can be compartmentalized which enables incubation of targets with fluorescent biomarkers because each droplet acts as a microscopic bioreactor^8–10^. Positive droplets will then light up under fluorescence excitation and can be detected with high signal-to-background ratio. Yet, fluorescence activated cell sorting (FACS), the most widely employed cell-sorting device, cannot sort (single emulsion) microdroplets which are often required for the identification of rare targets^11–13^. Although FACS droplet recovery has been demonstrated using double emulsified droplets, post-FACS analysis still showed a large fraction of ruptured droplets^14,15^. Non-destructive fluorescence activated droplet sorting (FADS) in a microfluidic device allows for downstream phenotypic and molecular analyses such as sequencing on positive droplets, which are not possible in the presence of large amounts of other biomaterials. However, microfluidic-based sorting ranging from active systems exploiting acoustic, electric, optical or magnetic forces, to passive systems relying on inertial forces or immobilization procedures are typically limited to small (< 1 mL) total handled sample volumes simply not suitable for rapid diagnostic assays on low-abundance pathogens, that require exploration of a 1–10 mL clinical sample just to identify sufficient targets for meaningful downstream analysis^11,16^. Particle enrichment in a microfluidic device is limited by fluid friction typically restricting sample flow rates to ~ 5 µL/min, not practical for many diagnostic applications that require multi-mL samples including BSIs^17^. Further, microfluidic devices are often expensive, fragile, and require specialized equipment and trained personnel for their operation disqualifying these devices from application in a clinical environment. Instead, our 3D particle sorting device scans a bulk sample at rates exceeding 400 µL/min by spinning a cylindrical, transparent sample vial in front of a laser focused to excite and detect target fluorescence. Moving the entire sample in a container in a helical motion avoids fluid flow restrictions and simplifies sample handling^18–20^. Limited to detection only, we have previously used this 3D particle counting technology to detect single bacteria of a specific species in whole blood using a droplet DNAzyme-based labeling approach^7^. More recently, we demonstrated bacterial antibiotic susceptibility testing in whole blood using one-step, high throughput blood digital PCR^21^. Here, we demonstrate that this principle is not limited to detection only, but that targets can also be sorted out in real time via a thin gauge needle or capillary dipping into the sample adjacent to the laser focus. We show that targets at concentrations of 1–100/mL can be enriched > 100-fold from 1 to 10 mL volumes within minutes to enable highly specific downstream analyses. In a proof-of-concept application, we demonstrate how bacteria in blood can be detected, isolated, identified, and tested for antibiotic susceptibility. Every year, sepsis as a result of bloodstream infection affects 30 million people worldwide, leading to an estimated 5 million deaths^22–24^. This high mortality rate is due in part to the inability to accurately diagnose underlying infections in the first hours of illness when treatment with antibiotics is most effective^6,25^. Further exacerbating these health concerns is the rise of antibiotic-resistant bacteria, presenting a major worldwide health threat. Also, BSIs associated with antibiotic resistance are often managed within intensive care units with high associated costs, imposing significant healthcare, economic, and social burdens^26^. Several studies have demonstrated that reduced time-to-result for bacterial ID/AST using rapid tests is directly correlated with reduced mortality rates and healthcare costs^27–31^. To address this issue, our workflow demonstration comprises upstream droplet encapsulation of the raw sample (Fig. 1a), detection and isolation of bacteria positive droplets within minutes by 3D particle sorting (Fig. 1b), and downstream sequencing and AST (Fig. 1c).

As a platform, 3D particle sorting could be broadly applied to resolve other pressing issues such as the isolation of viruses, circulating tumor cells, antibody-producing B cells and antigen-specific T cells, and HIV reservoir cells in blood. Even for applications where the high cost and difficulties with handling microfluidics is less problematic, such as the development of novel therapeutics that require isolation of highly specific targets, our approach could be highly beneficial. Ideally, screening for targets such as antibody-producing B cells should be feasible within a single working day of ~ 8 h, a major bottleneck that our 3D particle sorting approach is able to overcome.

## Results

### Principle of 3D particle sorting

To be able to screen large 1–10 mL sample volumes within a few minutes, a solution of highly diluted fluorescently labeled particles was placed into an optically transparent, cylindrical container and secured to a motorized rotary and axial translation stage. A laser beam was then focused into the container from the side via an objective lens to excite positive particles (Fig. 1d). From the focal spot, fluorescence signals were detected as a function of time with a photomultiplier (PMT) detector. To scan the 3D sample volume, the sample cuvette was rapidly rotated (~ 200 rpm) and slowly moved vertically (~ 1 mm/s) to create a spiral motion. If a positive particle passed the focal volume, the fluorescence signal was registered as a “hit” by real time analysis with a correlation filter. The number of hits over time can be converted to a concentration by referencing with a sample of known concentration. By moving the entire sample instead of pushing the fluid through a narrow microfluidic channel, limitations due to fluid friction can be avoided resulting in a very high sample throughput of ~ 0.4 mL/min (see Materials and Methods). To be able to not only detect but also extract positive particles, we added a sorting module to our setup. Real-time particle sorting was realized by dipping a thin gauge needle or capillary into the sample container with the tip at the same axial position shifted 90° along the radial trajectory of the laser focus. Hence, a particle passing the laser focus will reappear at the capillary tip after a certain time delay proportional to the rotation speed and the circumference of the scanning trajectory. Through applying suction by briefly opening a low latency solenoid valve connected to a vacuum system, a small fluid volume containing the positive particle could be extracted. Real time signal processing with a correlation filter was used to ensure that the signal shape matches the profile of positive particles and to enable a consistent time delay between particle detection and valve actuation (Supplementary Fig. S1). To ensure that the introduction of an extraction needle/capillary did not interfere with flow in a way that could negatively impact the detection efficiency, we calculated the Reynolds number, defined as $Re=\rho vL/\mu$, with $\mu$ the viscosity of the fluid, $v$ the flow speed, $\rho$ the density of the fluid, and $L$ the characteristic length. Turbulent flow typically occurs when $Re>2,900$, and laminar flow at $Re<2,300$. With $L$ equal to the cuvette diameter (1 cm), the parameters of water and a cuvette rotation speed of 200 rpm, we get a Reynolds number of $Re=1,170$ indicating perfectly laminar flow. We also experimentally verified that the introduction of a needle/capillary did not interfere with accurate particle detection. No significant differences were observed for particle detection with and without needle/capillary (Supplementary Fig. S2).

### Visualization of 3D particle extraction

**Figure 2:**
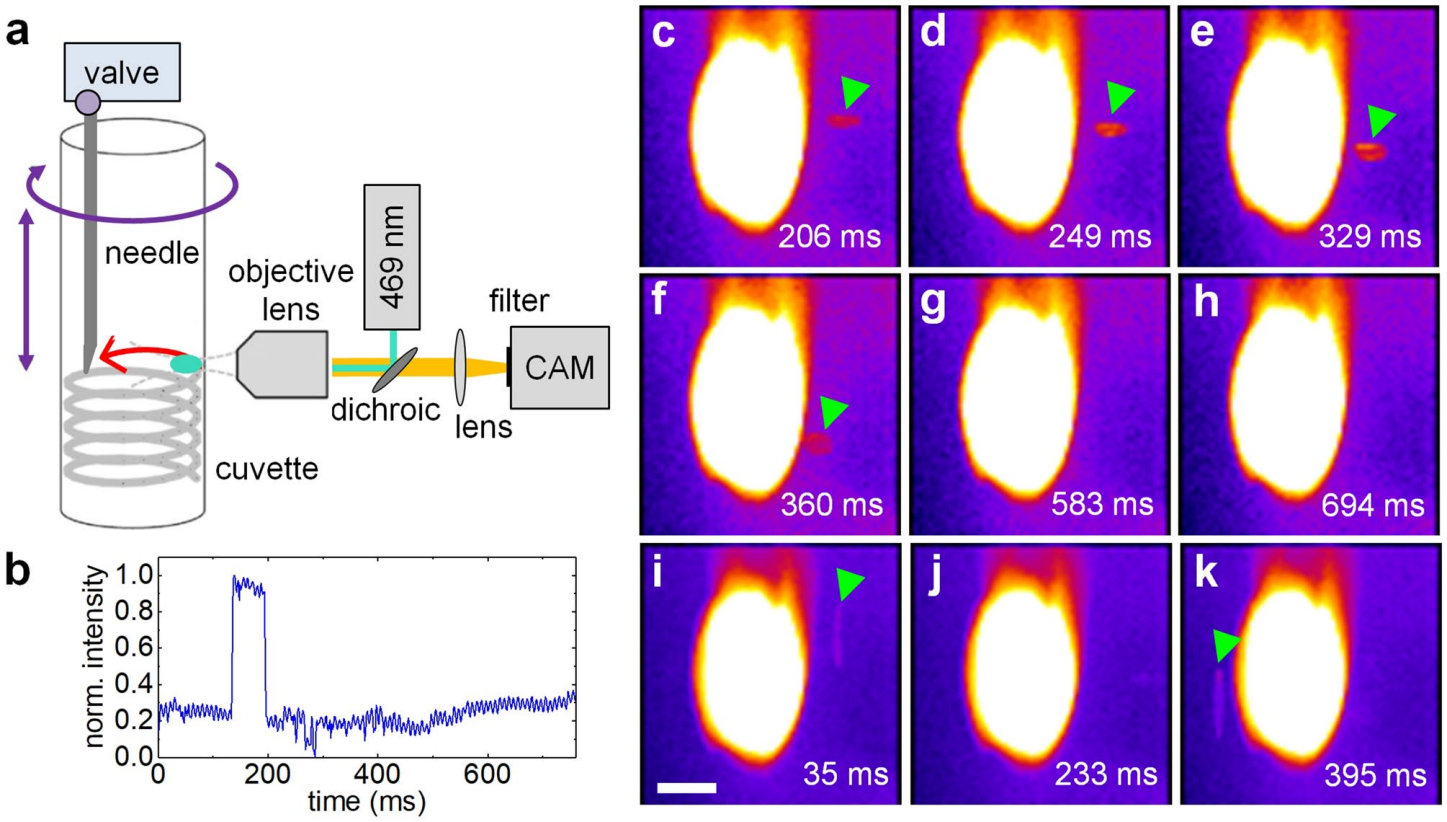
Visualization of particle extraction. (**a**) To verify the 3D particle sorting concept, image sequences were acquired during particle sorting at 630 fps with a camera replacing the PMT detector. (**b**) Sorting trigger signal with respect to the camera time sequence. (**c**–**h**) Exemplary frames of a positively triggered sorting event are shown. The needle tip was fluorescently labeled with Rhodamine 6G and appeared as a bright, saturated spot in the center of the images. A positive particle (indicated by arrows tips) entered the field of view from the right. In panels (**g**) and (**h**) it can be seen how the particle was aspirated into the needle and disappeared. (**i**–**k**) As a control, an event with the trigger signal deactivated shows that the positive particle reappears on the left side of the needle. We note that the cylindrical cuvette introduces some astigmatism and, as a result, the particles appear elongated in horizontal or vertical direction depending on their location with respect to the focal plane. To enable imaging, the cuvette rotation frequency was set to 25 rpm. Scale bar 100 µm.

To evaluate the 3D particle sorting concept, we replaced the PMT detector with a high speed camera to directly visualize particle uptake with a 30G needle (Fig. 2a, b).

A 3-mL solution of 15-µm yellow-green fluorescent beads at a concentration of 200 particles/mL was prepared and the extraction needle was fluorescently labeled by dipping the tip into a highly concentrated Rhodamine 6G solution. During the sorting procedure, images were continuously acquired at 630 Hz to allow direct visualization of fluorescent particles getting closer to the needle tip and eventually being aspirated into the needle itself. An exemplary, successfully triggered sorting event is shown in Fig. 2c–h. The stained needle tip appears as a stationary saturated fluorescent spot at the center of the field of view. From the image sequence, it can be seen how the particle entered the imaging area and, after valve activation at the appropriate time delay, disappeared when aspirated by the needle. The trigger signal with respect to the camera sequence is shown in Fig. 2b. As a control, the experiment was repeated while deactivating the extraction trigger signal (Fig. 2i–k). In the absence of a trigger signal, particles were not sorted and therefore reappeared on the other side of the needle. To facilitate imaging, the rotation speed of the cuvette was temporarily reduced from 200 to 25 rpm.

### Extraction and dispensing of droplets

**Figure 3:**
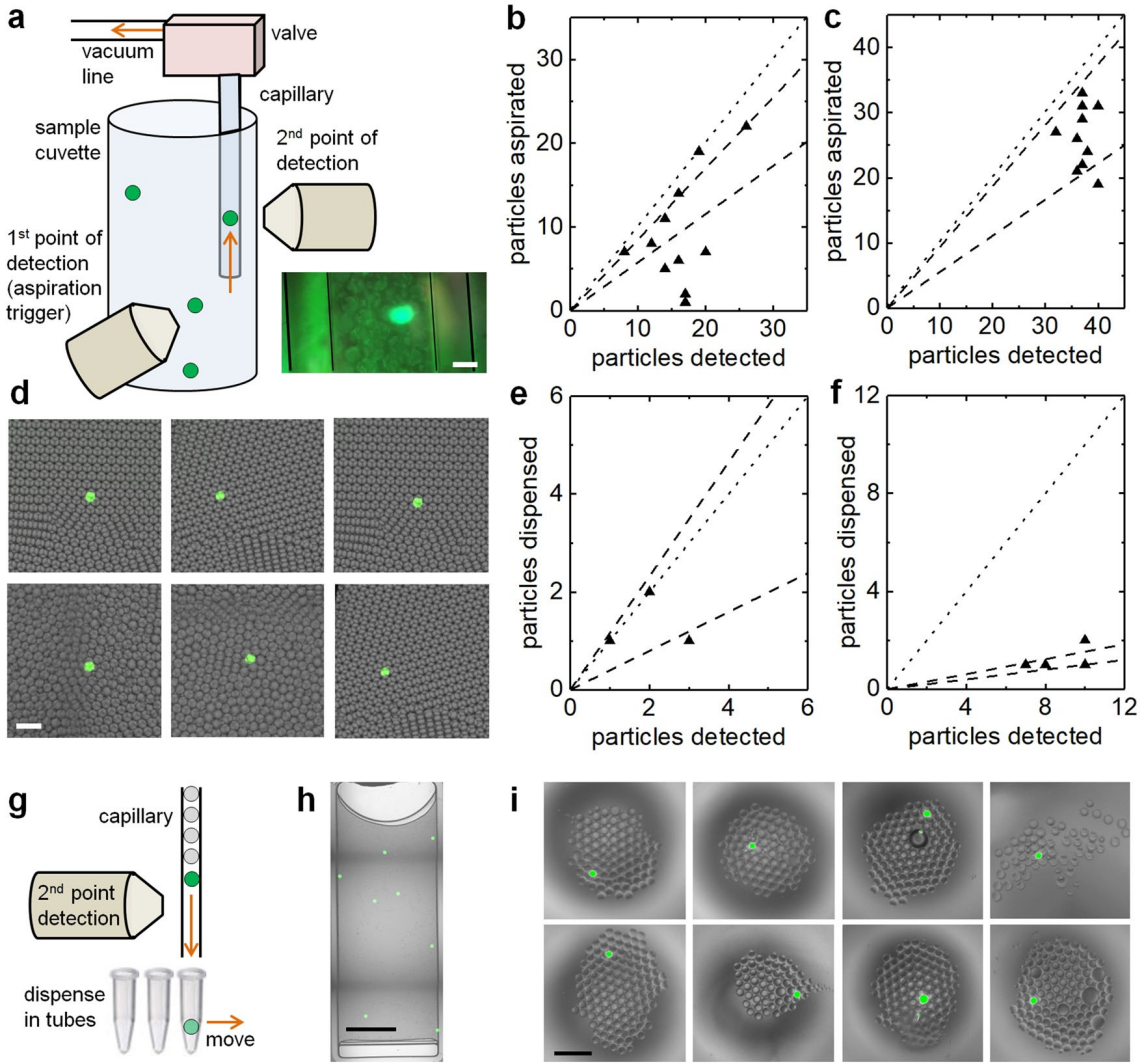
Quantification of 3D particle sorting and dispensing. (**a**) A second point of detection was focused into the capillary to compare particles numbers detected at the first point and sorted particle numbers The inset shows an image of a positive droplet aspirated into the capillary (scale bar, 100 µm). (**b**, **c**) The principle of this arrangement was first verified with fluorescent beads at 2,000 particles/mL, then with droplets at 500 positive droplets/mL. Plotted as triangles are the number of events detected in the capillary for each experiment as a function of the number of particle detected in the cuvette (N = 10, 11), the dashed lines represent the range of the mean ± standard deviation, the dotted lines represent 100% sorting efficiency. To quantify the dispensing efficiency, solutions of 50 positive droplets in 2 mL of negative droplets were prepared. After 3D sorting, the capillary was removed from the system and the content dispensed in microscope counting slides. (**d**) Exemplary, zoomed-in fluorescence images of dispensed positive particles in counting slides (scale bar, 300 µm). (**e**, **f**) Plotted as triangles are the number of positive particles found in the microscope counting slides after dispensing as a function of the number of detected particles at extraction volumes of 2.5 µL and 0.4 µL (N = 3, 4), the dashed lines represent the range of the mean ± standard deviation, the dotted lines represent 100% dispensing efficiency. (**g**) By selective dispensing the targets can be further enriched by actively dispensing the droplets passing through a transparent capillary with fluorescence excitation/detection near the tip. (**h**) Exemplary brightfield/fluorescence image overlay of a 10-μL volume of ~ 40,000 droplets with 8 targets containing 15-μm yellow-green fluorescent beads as typically obtained after 3D sorting. An exemplary volume was imaged inside a counting slide (scale bar, 5 mm). (**i**) Exemplary brightfield/fluorescence image overlays of targets dispensed into PCR tubes, yielding 1 target in 88 ± 23 droplets (mean ± SD, N = 8) (scale bar, 300 µm). Measurements with higher detection events at the capillary than at the first point of detection due to random sorting were disregarded as those would have falsely inflated the sorting efficiencies.

Next, we quantified the extraction efficiency with fluorescent beads and, more importantly, with droplets. For this purpose, we replicated the excitation/detection arm and mounted it to a xyz translation stage positioned at a 90° angle around the sample cuvette with respect to the optical axis of the original excitation/detection arm. We replaced the thin gauge needle with an optically transparent glass capillary and, after alignment, moved the second point of detection upwards by 0.5 mm to detect particles taken up into the capillary (Fig. 3a).

First, we prepared a 3-mL solution of 15-µm yellow-green fluorescent beads at a concentration of 2,000 particles/mL and subjected it to continuous detection/sorting runs of 30 s each. The number of detected particles in the cuvette and in the capillary were counted for each run and are plotted in Fig. 3b. The extraction efficiency (ratio of detected events capillary/cuvette) was 71 ± 14% (mean ± SD) with extracted volumes of 2.5 µL each. Second, we generated droplets (~ 70 µm diameter), as previously described^7^, filled with PBS buffer solution (negative) as well as droplets filled with 10 µM FITC dye solution (positive), prepared a mixture of ~ 500 positive droplets in 1 mL of negative droplets, and repeated the particle sorting experiment. An average extraction efficiency of 62 ± 33% (mean ± SD) for 2.5 µL extraction volumes was obtained, see Fig. 3c. We note that considering the standard deviations of the measurements (71 ± 14% and 62 ± 33%) the two averages are still within their respective ranges. Nevertheless, we presume that 15-µm beads in an aqueous solution are easier to aspirate due to their lower mass compared to larger 70-µm droplets. For these tests, particles were continuously extracted accumulating in the capillary, the tubing and, eventually, in the compensating reservoir. However, the goal of rapid, 3D particle sorting is to dispense the particle-enriched volumes into aliquots suitable for downstream analyses such as sequencing. To demonstrate and verify dispensing, we prepared a mixture of ~ 50 dyed droplets in 1 mL of unlabeled droplets for particle sorting. We stopped each sorting run after a few particles were detected, detached the capillary from the system, dispensed the contents into microscope counting slides, subjected them to fluorescence imaging (Fig. 3d), and counted the number of positive particles. From the image data, extraction-dispensing efficiencies of 78 ± 39% (mean ± SD) were found for extraction volumes of 2.5 µL (Fig. 3e). When reducing the extraction volume to 0.4 µL per event, the extraction efficiency dropped to 14 ± 4% (mean ± SD) (Fig. 3f). In our current 3D sorter prototype, the aspiration volume is determined by vacuum level and activation time of the solenoid valve, lacking precision to extract sub-microliter volumes with high efficiency. In the future, we intend to precisely control the aspiration volume by addition of a flow sensor. However, instead or in addition to the reduction of the aspiration volume, targets can be further enriched by selective dispensing (Fig. 3g). To illustrate this strategy, we dispensed a 10-µL volume (~ 40,000 empty droplets) containing 8 targets (droplets with 15-µm yellow-green beads), as typically obtained after 100-fold enrichment by 3D particle sorting, at a rate of 1 µL/min (Fig. 3g, h). For this purpose, we loaded the sample in a syringe coupled to a 30 cm long glass capillary of 100 µm inner diameter through which the sample was pushed with a syringe pump. The capillary passed a second point of detection with simplified, threshold based detection of positive droplets. Upon detection of a positive droplet, dispensing was triggered by moving a plate with PCR tubes into position with a motorized xyz stage. The dispensing setup and example images of droplets dispensed into PCR tubes are shown in Supplementary Fig. s3. By hovering the capillary over a waste tube until a positive droplet was detected near the tip, followed by moving to a target container and dipping the end of the capillary for 3 s to dispense the positive droplet, we were able to further enrich samples for positive droplets. Images of each target tube confirmed the presence of positive droplets, now enriched to a ratio of 1 in 88 ± 23 empty droplets (mean ± SD) (Fig. 3i). With this selective dispensing strategy, each positive droplets was contained in only ~ 26 nL of negative droplets corresponding to another 60-fold target enrichment to possibly reach a > 10,000-fold total enrichment.

### Isolation of bacteria directly from blood

**Figure 4:**
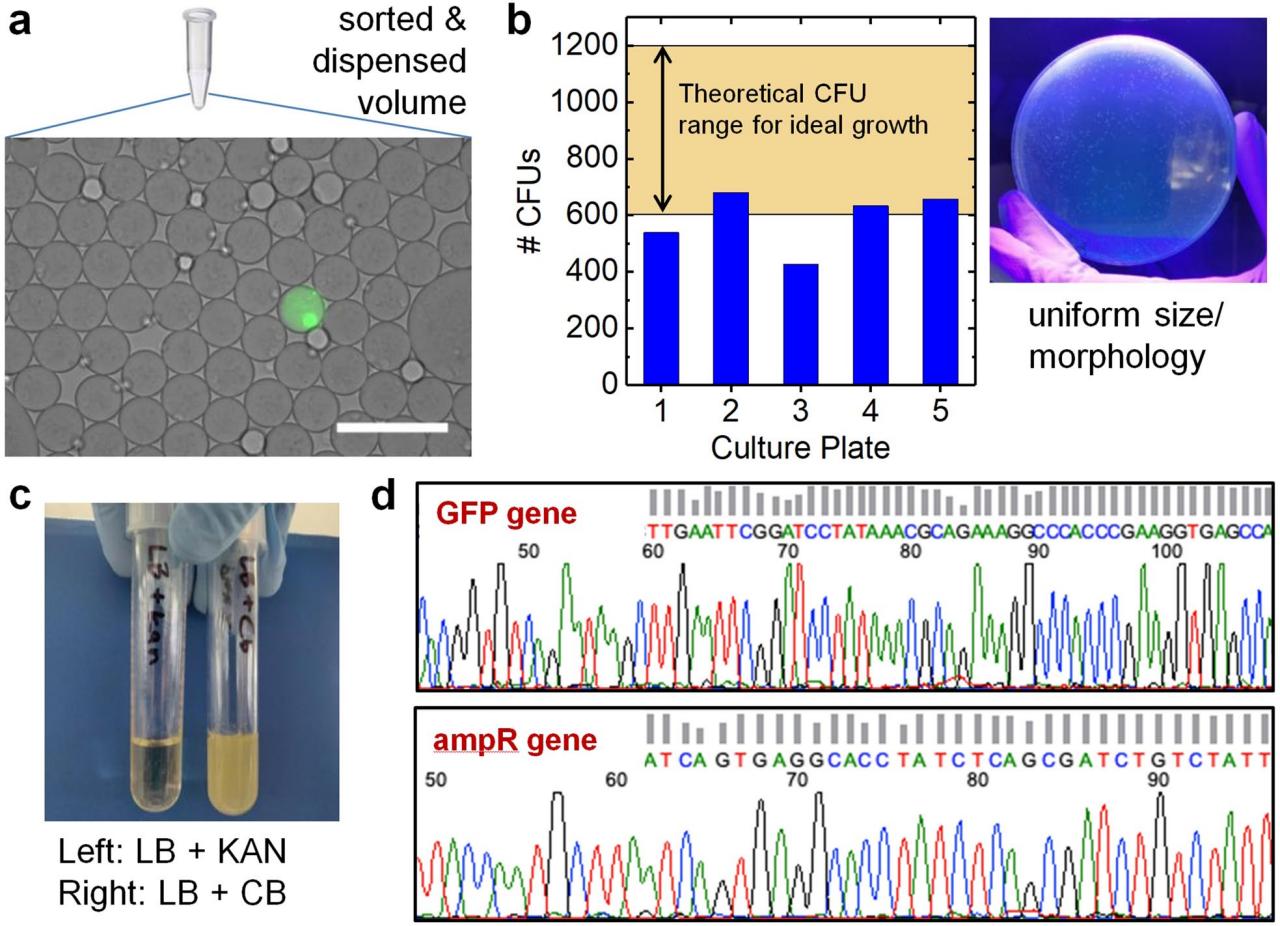
Workflow demonstration of bacteria sorting in whole blood. Blood of healthy donors was spiked with carbenicillin resistant *E. coli* expressing GFP, encapsulated in droplets, and subjected to 3D particle sorting. (**a**) Positively triggered, single volumes were extracted and dispensed into microscope counting slides to verify the presence of a single, positive droplet. (**b**) Five extracted volumes were dispensed into individual tubes and subjected to overnight plating. The number of colonies matched the theoretical growth expectation across all five replicates (100% accuracy), and the uniform size/morphology indicated monoclonal origin from single positive droplets. (**c**) Growth in medium with antibiotics confirmed kanamycin sensitivity (left) and carbenicillin resistance (right). (**d**) Snippets of sequencing chromatography confirming the presence of *gfp* (top) and antibiotic resistance (*amp*) regions (bottom), the full sequences are shown in the Supporting Information. Scale bar 200 µm.

After verification and quantification of the 3D particle sorter, we selected a proof-of-concept application in which we aimed at sorting out bacteria from whole blood for downstream bacterial ID and antibiotic susceptibility testing as demanded for diagnosis of sepsis. The biggest technical challenge associated with current systems is their inability to rapidly detect pathogens that usually occur at low concentrations, i.e., < 1–100 colony-forming units (CFUs) per milliliter of blood. Existing phenotypic methods typically involve an initial culture step (12–72 h) to grow bacteria, followed by bacterial ID and AST (4–24 h), requiring several days to result, which is too slow to allow physicians to make timely treatment decisions^3^. Molecular diagnostic methods including PCR, nanoparticle-based assays, and sequencing methods can reduce assay time but are often not sensitive enough, therefore still requiring lengthy culture-enrichment periods^2,4,5^. Instead, our 3D particle sorting technology can uniquely address the major unmet need of sorting out low-abundance bacteria directly from large volume blood samples minimizing and possibly eliminating the need for culture enrichment. In a proof-of-concept study, we spiked carbenicillin resistant but kanamycin susceptible *E. coli* expressing green fluorescent protein (GFP) into a blood sample, followed by droplet encapsulation, and droplet sorting with the high-throughput 3D particle sorter. The detection of a positive droplet triggered a single sorting event extracting 5 µL of sample. To verify that the extracted volumes contained droplets with bacteria, we dispensed the volumes of four independently triggered sorting events into microscope counting slides, an exemplary, zoomed-in image of a single, positive droplet is shown in Fig. 4a.

From another sample, five volumes were independently extracted, dispensed and subjected to overnight plating. The number of colonies matched the theoretical ideal growth expectation across all five replicates (100% accuracy), and the uniform size and morphology indicated monoclonal origin from single positive droplets (Fig. 4b). Further, growth in medium with antibiotics confirmed carbenicillin resistance and kanamycin susceptibility (Fig. 4c), and Sanger sequencing confirmed the presence of *gfp* and the antibiotic resistance (*amp*) markers (Fig. 4d, Supplementary Figs. S4–S8). These results strongly support the proposed workflow from particle detection in whole blood to isolation of positives, and downstream molecular and phenotypic analyses. We note that, for proof-of-concept, we utilized GFP-expressing bacteria to provide a distinct target sequence for downstream analysis. To exclude any labeling-related false positive or false negative events, droplets were incubated over night to enhance the fluorescent signal from GFP-expressing bacteria. Of course, for the detection of bacteria in clinical blood samples, which lack expression of a fluorescent marker, the sample must be encapsulated in droplets together with suitable biomarkers. In Supplementary Fig. S9, we show how bacteria-containing droplets can be rendered fluorescent by sample encapsulation with SYBR Green I, an asymmetrical cyanine dye used as a nucleic acid stain in molecular biology. Cell secretion might also be utilized for fluorescence signal generation in droplets. As an example, we encapsulated *E. coli* with the β-lactamase sensor Fluorocillin in Supplementary Fig. S10. Another popular approach includes the usage of metabolic dyes as reporters including resazurin, a blue non-fluorescent dye that is reduced to the pink and highly fluorescent compound resorufin by metabolically active bacteria^32–35^. We previously demonstrated that droplet encapsulation of ~ 2 mL clinical blood samples for sepsis diagnosis can be achieved within ~ 40 min with a high throughput microfluidic droplet generator with 4 parallel nozzles^21^. Based on fluorescence labeling assays of bacteria-containing droplets as shown in Supplementary Fig. S9, we expect an optimized labeling procedure to take < 5 h. Sorting and dispensing only takes ~ 20 min resulting in a total assay time of < 6 h from raw blood to sorted single bacteria which is significantly faster than a 24–72 h blood culture enrichment. Current clinical bacterial ID and AST assays take an additional 4–24 h after either 3D sorting or blood culture. While initial diagnosis of sepsis would benefit from further reducing time to bacteria detection, AST can tolerate a few hours of wait time to correct initial treatment with more precision antibiotic information^36^. In future applications, novel single cell downstream AST methods could help to further reduce the time-to-result. Suitable methods include multiplex TaqMan PCR for specific antibiotic resistance genes for a target panel of the most prevalent pathogens and next generation sequencing technologies such as MinION nanopore sequencing that offer real-time sequencing to rapidly identify bacterial ID and drug resistance profiles^37–40^. Single cell AST could also elucidate variability in susceptibility phenotypes not often detectable and discernable in bulk populations^41,42^.

## Discussion

The 3D particle sorting technology can uniquely address the major unmet need for detection and isolation of rare particles (1–100 per mL) directly from larger, clinically relevant fluid volumes (1–10 mL) within minutes. While existing biochemical or microfluidic methods lack sensitivity or take many hours to days to detect highly dilute, single targets, 3D particle sorting optically scans bulk, unprocessed samples at rates exceeding 0.5 mL/min by spinning a cylindrical sample vial in front of a detector not limited by fluid friction as in (micro)fluidic devices that are restricted to ~ 0.005 mL/min. At the same time, real time data processing allows for aspiration via a thin gauge needle or capillary to extract positive targets with high efficiency. Such enrichment by particle isolation eliminates the need for lengthy sample processing such as culture and biochemical extraction substantially, reducing the time-to-result. In combination with sample encapsulation in microdroplets together with fluorescent biomarkers to specifically activate fluorescence in positive droplets, 3D particle sorting presents a very powerful technology for diagnostic applications. Here, we have demonstrated the utility of this approach towards sepsis diagnosis by isolation of bacteria-containing droplets from blood samples. FACS, the current gold standard for cell sorting, currently cannot sort single emulsion droplets. While FACS of double emulsified droplets has been demonstrated, generating double emulsified droplets is difficult and FACS efficiency is still not optimal due to frequent droplet rupture during sorting^14,15^. While FACS may further improve in the future, we believe our 3D particle sorting technology to be advantageous for applications where very few particles (1–10/mL) need to be detected. For ultralow concentrations, our approach can provide superior speed and is compatible with well established droplet generation methods. Currently, droplets could be extracted reliably (78% efficiency defined as number positive droplets extracted per number of droplets detected) with extraction volumes of 2.5 µL, which corresponds to a 400-fold enrichment considering a single positive particle contained in 1 mL of fluid. In the future, we aim at reducing the extraction volume to the sub-microliter regime using a flow sensor and to couple extraction with automated, selective dispensing via a second point of detection with the goal of reaching a > 10,000-fold enrichment. Also, we will continue to develop droplet-based labeling strategies utilizing different biomarkers to be able to fluorescently light up a wide variety of targets^7,21^. For downstream analysis of sorted targets, novel sequencing technologies such as real-time MinION nanopore sequencing could rapidly identify targets^37–40^. For example, a recent study reported that MinION nanopore sequencing can identify bacterial species and strain information within 1 h of sequencing time and initial drug resistance profiles in 2 h^37^. In summary, offering a 100-fold increase in sample processing speed with single particle sensitivity in complex biological fluids including blood, we expect our 3D particle sorting technology to be capable of detecting and characterizing pathogens such as bacteria and viruses, circulating tumor cells, target immune cells (e.g., neoantigen-specific T cells, antibody-producing B cells) from their repertoire, and HIV reservoir cells with the potential of saving many lives across the globe.

## Methods

### The 3D particle sorting setup

The 3D particle sorter was based on our previously published 3D particle counter^7,18^. Briefly, the sample (0.5–3 mL) was filled into a cylindrical glass vial (Abbott, IL, USA) of 10 mm diameter and 50 mm height. With motorized rotation and z translation stages, the cuvette was spun (200 rpm) and moved up and down (1 mm/s) in front of a 20 ×, NA 0.4, 9 mm focal length objective lens (M-20X, Newport, CA, USA). A beam from a 469-nm diode laser (Model 73225, ISS, IL, USA) was cleaned up with a 465/30 nm band pass filter (FF01-465/30, Semrock, NY, USA), reflected off a 495 nm long pass dichroic mirror (FF495-Di03, Semrock), and focused into the cuvette via the objective lens. Fluorescence was collected with the same lens, filtered with a 535/50 nm band pass filter (FF01-535/50, Semrock), and focused onto an aperture of 500 µm diameter to reject out-of-focus light before detection with a photomultiplier (H9305-04, Hamamatsu, Japan). All optics were mounted on a motorized translation stage such that the position of the focal volume inside the sample cuvette could be adjusted. This excitation/detection arm was replicated and installed at a 90° angle around the rotation axis of the cuvette with respect to the first point of detection. Mounted on a manual xyz translation stage, the second point of detection was used to determine the trajectory of the first point of detection for precise suction needle/capillary positioning and, at a position 0.5 mm above the trajectory, used to count particles entering the suction capillary. This second detection point can also be used for active dispensing of positive droplets for further target enrichment. At high particle concentrations, it is possible that more than one particle is extracted per positive trigger event. Those points with higher detection events at the capillary than the first point of detection were disregarded for calculation of extraction efficiencies. For signal acquisition, an analog-to-digital converter card was used (DaqBoard 3000, IOTech Inc., OH, USA). In-house software based on applying a correlation filter in real-time to detect spikes in the PMT signal corresponding to positive particle passing the detection volume was used to detect particles and to trigger the suction signal at an appropriate delay (75 ms). As the width of positive peaks in the signal is proportional to the particle size and the time it takes for each particle to cross the excitation volume, particles can be counted by applying a Gaussian pattern recognition filter with matching standard deviation (sigma). The sigma value has to be determined before sorting, however, does not change between experiments if the same rotation speed and target particle size are used. Hence, a single calibration run with a reference sample any time prior to particle sorting is sufficient. For the initial, camera-based verification of the 3D particle sorting principle, the PMT in the second detection arm was replaced with a high speed EMCCD camera (Photometrics Cascade 128+, Photometrics, USA) running at 630 fps to visualize particle extraction in real time. The valve trigger signal was recorded by positioning a green LED close to the camera chip. When a particle was detected, a trigger signal was sent to both activate suction and to turn on the green LED to visualize the trigger event in the camera images.

### Particle extraction

The fluidic part of the 3D particle sorter consisted of a low latency solenoid valve (Type 6724, Buerkert, Germany) connected to a vacuum line via a compensating reservoir filled with water using PEEK tubing (OD 1.5 mm, ID 0.1 mm, IDEX Health & Science, USA). The valve inlet was directly connected to exchangeable capillaries of 20 µL volume (440 µm inner diameter, 125 mm length) (21–164-2D, ThermoFisher Scientific, MA, USA). Suction volumes were adjusted to 0.4–5 µL per trigger event by a variable bypass in the vacuum line and by opening the valve for durations of 5–20 ms. After triggering a positive event, a lockout period of 200 ms was applied to ensure single suction events. The valve with attached suction capillary was mounted on a xyz manual translation stage and 10-µm precision micrometers were used to precisely position the needle/capillary within the sample cuvette. For the initial, camera-based verification of the 3D particle sorting principle, a programmable microinjector (IM-300, Narishige, Japan) connected to the vacuum line was used instead of the solenoid valve to extract particles. Targets in enriched, 10-μL volumes were actively dispensed by passing the droplets through a transparent capillary with fluorescence excitation/detection near the tip. To generate a stable flow of 1 μL/min, a syringe pump (NE-500, New Era) was used but a pressure system could be used as well. To switch between waste tube and target tubes, a motorized xyz stage (BCH40-10, Barch) was used. Target tube dipping time was 3 s.

### Sample throughput estimation

The sample throughput of the 3D particle sorter was given by the size of the detection volume, the rotation speed of the cuvette, and the radius of the scanning trajectory. The focal volume, ${V}_{det}$, can be estimated as

1 $${V}_{det}={\frac{\pi }{2}}^{3/2}{\omega }_{x}{\omega }_{y}{\omega }_{z}$$

where $\omega$ is the waist of a 3D Gaussian in x,y and z. Given the optics of our system, we got ${\omega }_{x}\sim 70\,\upmu\text{m}$, ${\omega }_{y}\sim 500\,\upmu\text{m}$, and ${\omega }_{z}\sim 150\,\upmu\text{m}$, resulting in an observation volume size of ~ 5 nL. The scanned volume can then be calculated as

2 $${V}_{scan}=\frac{\pi }{2}{\omega }_{y}{\omega }_{z}2\pi frt$$

with cuvette rotation frequency, $f$, scanning trajectory radius, $r$, and scanning time, $t$. Given a rotation frequency of 200 rpm, a scanning radius of 4 mm (5 mm cuvette radius and 1 mm wall thickness) by placing the focal volume close to the cuvette wall, we estimated the scanned volume as ~ 0.4 mL/min. We note that, in the 3D particle sorter, the sample scanning is a stochastic process as the same sub-volume might be scanned twice while other sub-volumes of the cuvette might not be scanned at all, especially near the rotation axis, where the laser focus does not reach. Exploration of the entire volume is helped by mixing of the sample during the scanning process. However, when particle sorting is activated, each particle will be counted only once as it is being extracted immediately.

### Sample preparation

For various tests of the 3D particle sorter, 15 µm yellow-green fluorescent beads (Fluorospheres, F8844, Invitrogen) were serially diluted to reach the desired target concentrations. Negative droplets filled with PBS buffer and positive droplets filled with FITC dye solution were generated as previously described^7^. Blood samples used in this study were obtained with informed consent from healthy donors and approval from the Institutional Review Board (IRB 2012-9023) via the Blood Donor Program at the UCI Institute for Clinical and Translational Science (ICTS). Samples for the bacteria sorting from blood proof-of-concept study were prepared by spiking GFP-expressing *E. coli* (*gfp cbampr*) at 5,000 CFU/mL into a 50/50 LB/isotonic solution + 4% glycerol + 10% whole blood and encapsulating into droplets (average 90 µm diameter, 382 pL volume) plus carbenicillin followed by overnight culture at 37 °C in an aerobic environment.

### Blood samples ethical approval

De-identified blood samples from healthy donors used in this study were obtained with informed consent via the Blood Donor Program at the UCI Institute for Clinical and Translational Science (ICTS). The study was approved by the UC Irvine Office of Research Institutional Review Board under UCI IRB HS# 2012-9023. All experiments were carried out in accordance with relevant guidelines and regulations.

### Bacteria culture, sequencing and AST

Single dispensed volumes from each of the 5 independent trigger events were individually spiked into 100 µL of fresh LB growth media and then plated on solid agar plates. LB agar plates were made in standard 9 cm diameter Petri dishes. Each sample was spread (0.10 mL) onto an independent agar plate using a sterile loop and turntable. All plates were incubated overnight at 37 °C and colonies were counted the following morning. From each culture plate, 7 colonies were selected and inoculated in 3 mL liquid LB media under selective pressure with 100 µg/mL carbenicillin or kanamycin and incubated overnight at 37 °C to demonstrate the phenotypic antibiotic resistance profile of the sorted organism. Additionally, isolated colonies from each culture plate were harvested for DNA extraction; DNA was sent to a reference lab for Sanger sequencing. Sequencing results were analyzed with ApE v2.0.60 software by running alignments against reference sequences for *gfp* and *amp* genes. Sequence chromatograms and alignments confirmed the specific presence of *gfp* and *amp* molecular targets in all sorted samples.

## Supplementary information

Supplementary Information.

## Data Availability

Upon request, we will make the data available to other researchers. The correlation filter routine for particle detection in fluorescence time traces is included in simFCS software available at https://www.lfd.uci.edu/globals/.
