# Supplementary material for: Rapid isolation of rare targets from large fluid volumes
Source: Sci Rep. 2020 Jul 27;10:12458. doi: 10.1038/s41598-020-69315-1 (PMC7385493; doi:10.1038/s41598-020-69315-1)
Supplement: Supplementary file 1 — Supplementary Information. [file 41598_2020_69315_MOESM1_ESM.pdf]

Rapid Isolation of Rare Targets from Large Fluid Volumes

Per Niklas Hedde[1,2,*], Margaux Bouzin[1,3], Timothy J. Abram[4], Xiaoming Chen[5], Melody N. Toosky[4], Tam Vu[5], Yiyan Li[6], Weian Zhao[1,5,7-10] & Enrico Gratton[1]

[1]Department of Biomedical Engineering, University of California, Irvine, CA, USA.

[2]Department of Biochemistry, University of Hawaii at Manoa, HI, USA.

[3]Physics Department, Università degli Studi di Milano-Bicocca, Milan, Italy.

[4]Velox Biosystems, Irvine, CA, USA.

[5]Department of Pharmaceutical Sciences, University of California, Irvine, Irvine, CA, USA.

[6]Department of Physics and Engineering, Fort Lewis College, Durango, CO, USA.

[7]Sue and Bill Gross Stem Cell Research Center, University of California, Irvine, Irvine, CA, USA.

[8]Chao Family Comprehensive Cancer Center, University of California, Irvine, Irvine, CA, USA.

[9]Edwards Life Sciences Center for Advanced Cardiovascular Technology, University of California, Irvine, Irvine, CA, USA.

[10]Department of Biological Chemistry, University of California, Irvine, Irvine, CA, USA.

These authors contributed equally: Per Niklas Hedde, Margaux Bouzin

[*]Correspondence should be addressed to phedde@uci.edu

Supporting Information

Supplementary Fig. S1	Aspiration timing verification.
Supplementary Fig. S2	Verification of particle detection after introduction of the aspiration needle/capillary.
Supplementary Fig. S3	Schematic and example images of droplet dispensing.
Supplementary Fig. S4	Sanger sequencing results of colony 1, sequence alignment to *sfgfp*.
Supplementary Fig. S5	Sanger sequencing results of colony 2, sequence alignment to *sfgfp*.
Supplementary Fig. S6	Sanger sequencing results of colony 3, sequence alignment to *sfgfp*.
Supplementary Fig. S7	Sequencing chromatograph results of validation of the gfp gene.
Supplementary Fig. S8	Sequencing chromatograph results of validation of the *amp* gene.
Supplementary Fig. S9	Fluorescence images of bacteria-containing blood after droplet encapsulation with SYBR Green I.
Supplementary Fig. S10	Encapsulation of *E. coli* bacteria with the β-lactamase sensor Fluorocillin.

Supplementary Fig. S1.

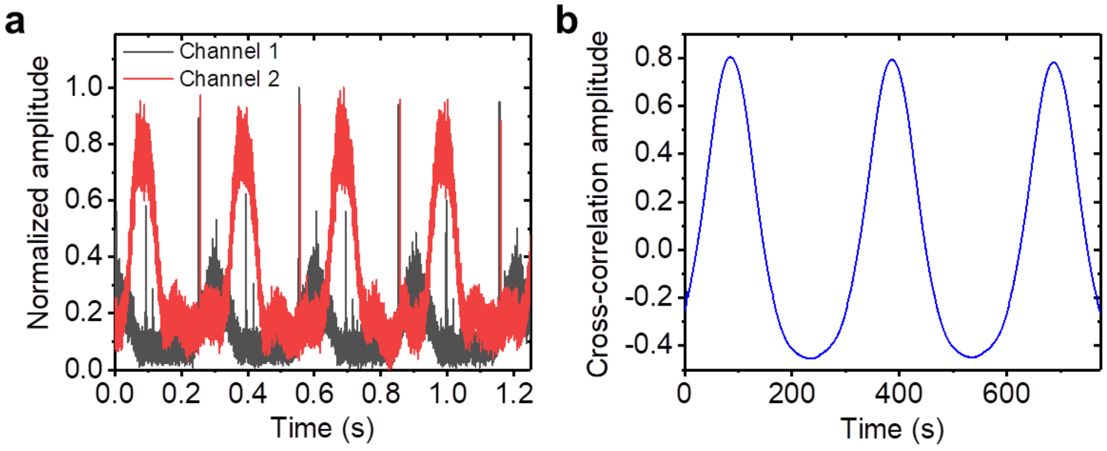

Aspiration timing verification. To be able to successfully extract particles, the suction needle position must be precisely aligned with the trajectory of the laser focus in the sample cuvette. For this purpose, we replicated the excitation/detection arm and mounted it to a xyz translation stage positioned at a 90º angle around the sample cuvette with respect to the optical axis of the original excitation/detection arm. To verify timing between first detection and reappearance of positive particles at the aspiration point, scattered light was detected off an empty glass cuvette. (**a**) The cuvette was spun at 200 rpm, portions of the time traces recorded in both channels are shown. The low frequency periodic signal variation was a consequence of a slightly eccentric cuvette movement due to minimal misalignment with respect to the rotation axis. The sharp, high frequency spikes can be attributed to increased scatter from dust or scratches in the cuvette surface. (**b**) Time traces recorded in both channels were cross-correlated. The cross-correlation maximum appeared at around 80 ms delay similar to the expected value of 75 ms at a phase shift of 90º at 200 rpm. We then positioned the extraction needle with a xyz translation stage at the position of the second laser focus and connected it to our vacuum system.

Supplementary Fig. S2.

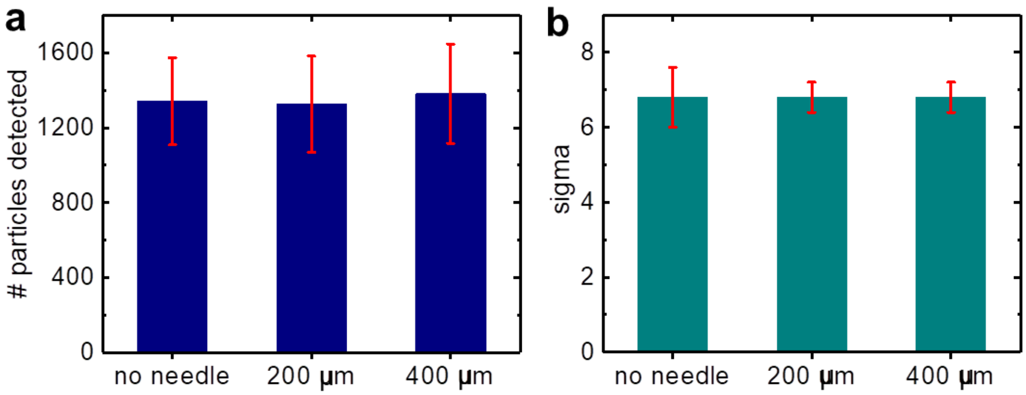

Verification of particle detection after introduction of the aspiration needle/capillary. To verify that the needle or capillary used to extract particles does not impede particle detection, a solution of 1-µm fluorescent beads was prepared and subjected to particle counting without and with needles of different thicknesses. No significant difference in (**a**) the number of detected particles and (**b**) in the optimal standard deviation for the Gaussian pattern recognition filter was observed. Error bars indicate the standard deviation over N = 6 one-minute long intensity time traces.

Supplementary Fig. S3.

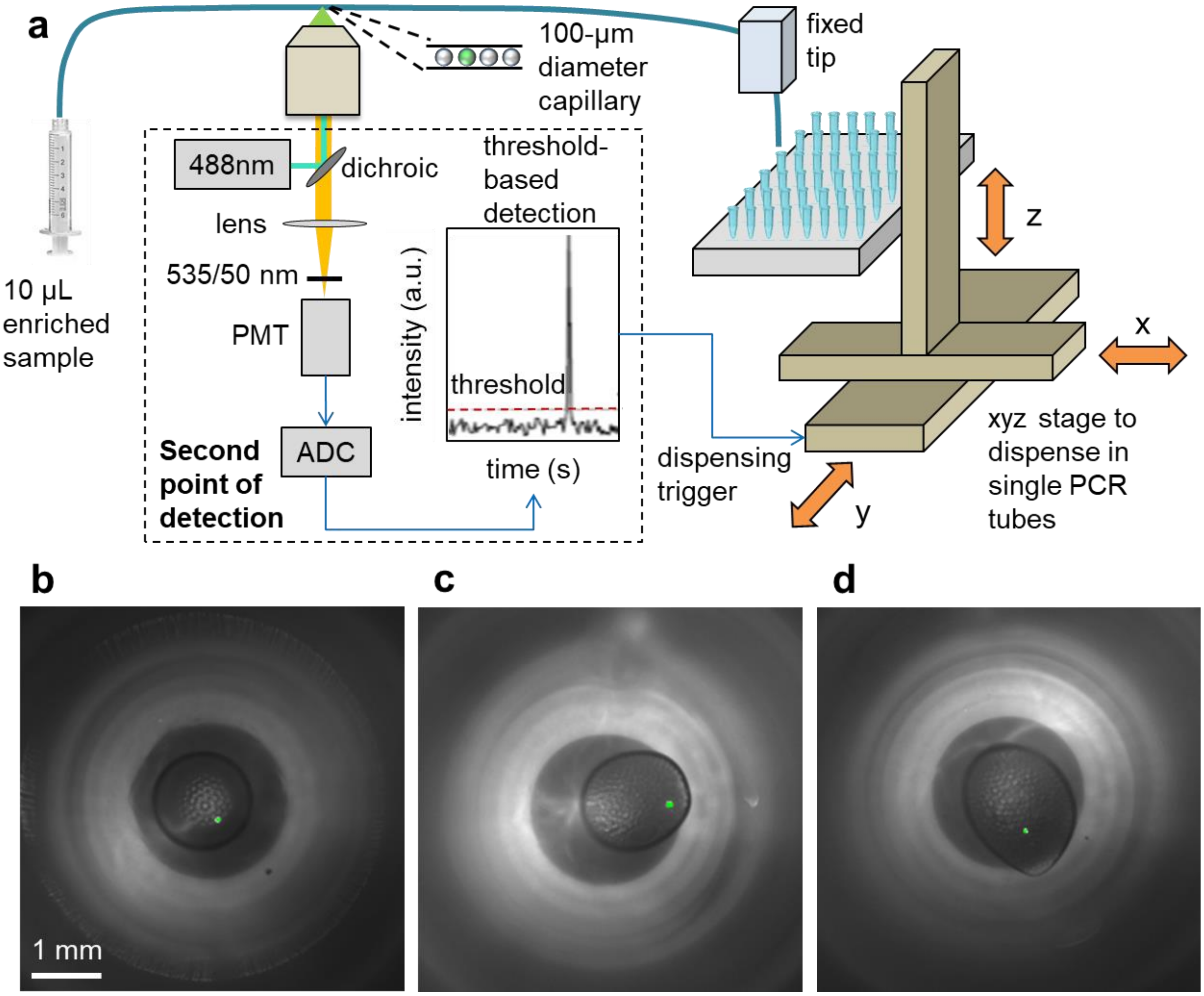

Schematic and example images of droplet dispensing. (**a**) Schematic of active dispensing. 10-µL volumes enriched for targets were passed through a 30 cm long glass capillary (ID: 100 µm, OD: 300 µm) with a syringe pump (NE-500, New Era) at 1 µL/min. Fluorescence was excited with a 488-nm laser at a distance of 10 cm to the capillary tip. When the signal passed a threshold indicating the presence of a positive droplet at the detection spot, a PCR tube was moved into position with a motorized xyz stage (BCH40-10, Barch) for droplet dispensing 5 s after detection. For dispensing, the capillary tip was dipped into the target tube for 3 s. (**b-d**) Example images taken with a camera coupled to a 4x objective lens from the top of PCR tubes after dispensing. Fluorescence was excited with a 488-nm to highlight positive droplets.

Supplementary Fig. S4.

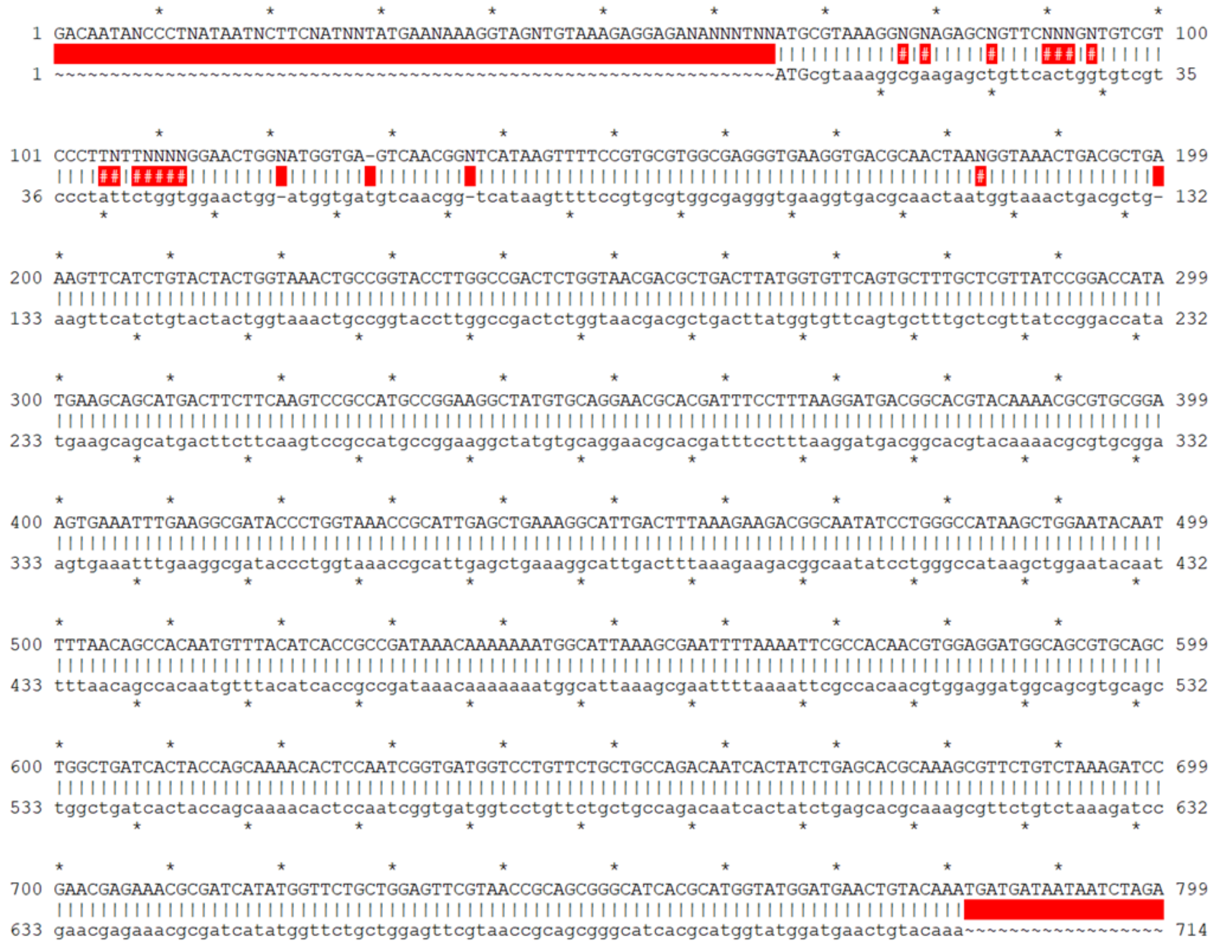

Sanger sequencing results of colony 1, sequence alignment to *sfgfp*. Matches: 698, Mismatches: 15, Gaps: 290, Unattempted: 0.

Supplementary Fig. S5.

```
             *         *         *         *         *         *         *         *         *         *
101 TGCTTCAATTAATNTGAAAAAGGTAGAGTAAAGAGGAGAAATACTNGATGCGTAAAGGCGAAGAGCTGTTCACTGGTGTCGTCCNTATTCTGGTGGAACT 200
                                                   |||||||||||||||||||||||||||||||||||||#|||||||||||||||
  1 ~~~~~~~~~~~~~~~~~~~~~~~~~~~~~~~~~~~~~~~~~~~~~~~ATGcgtaaaggcgaagagctgttcactggtgtcgtccctattctggtggaact 53
                                                            *         *         *         *         *

             *         *         *         *         *         *         *         *         *         *
201 GGATGGTGATGTCAACGGTCATAAGTTTTCCGTGCGTGGCGAGGGTGAAGGTGACGCAACTAATGGTAAACTGACGCTGAAGTTCATCTGTACTACTGGT 300
    ||||||||||||||||||||||||||||||||||||||||||||||||||||||||||||||||||||||||||||||||||||||||||||||||||||
 54 ggatggtgatgtcaacggtcataagttttccgtgcgtggcgagggtgaaggtgacgcaactaatggtaaactgacgctgaagttcatctgtactactggt 153
          *         *         *         *         *         *         *         *         *         *

             *         *         *         *         *         *         *         *         *         *
301 AAACTGCCGGTACCTTGGCCGACTCTGGTAACGACGCTGACTTATGGTGTTCAGTGCTTTGCTCGTTATCCGGACCATATGAAGCAGCATGACTTCTTCA 400
    ||||||||||||||||||||||||||||||||||||||||||||||||||||||||||||||||||||||||||||||||||||||||||||||||||||
154 aaactgccggtaccttggccgactctggtaacgacgctgacttatggtgttcagtgctttgctcgttatccggaccatatgaagcagcatgacttcttca 253
          *         *         *         *         *         *         *         *         *         *

             *         *         *         *         *         *         *         *         *         *
401 AGTCCGCCATGCCGGAAGGCTATGTGCAGGAACGCACGATTTCCTTTAAGGATGACGGCACGTACAAAACGCGTGCGGAAGTGAAATTTGAAGGCGATAC 500
    ||||||||||||||||||||||||||||||||||||||||||||||||||||||||||||||||||||||||||||||||||||||||||||||||||||
254 agtccgccatgccggaaggctatgtgcaggaacgcacgatttcctttaaggatgacggcacgtacaaaacgcgtgcggaagtgaaatttgaaggcgatac 353
          *         *         *         *         *         *         *         *         *         *

             *         *         *         *         *         *         *         *         *         *
501 CCTGGTAAACCGCATTGAGCTGAAAGGCATTGACTTTAAAGAAGACGGCAATATCCTGGGCCATAAGCTGGAATACAATTTTAACAGCCACAATGTTTAC 600
    ||||||||||||||||||||||||||||||||||||||||||||||||||||||||||||||||||||||||||||||||||||||||||||||||||||
354 cctggtaaaccgcattgagctgaaaggcattgactttaaagaagacggcaatatcctgggccataagctggaatacaattttaacagccacaatgtttac 453
          *         *         *         *         *         *         *         *         *         *

             *         *         *         *         *         *         *         *         *         *
601 ATCACCGCCGATAAACAAAAAAATGGCATTAAAGCGAATTTTAAAATTCGCCACAACGTGGAGGATGGCAGCGTGCAGCTGGCTGATCACTACCAGCAAA 700
    ||||||||||||||||||||||||||||||||||||||||||||||||||||||||||||||||||||||||||||||||||||||||||||||||||||
454 atcaccgccgataaacaaaaaaatggcattaaagcgaattttaaaattcgccacaacgtggaggatggcagcgtgcagctggctgatcactaccagcaaa 553
          *         *         *         *         *         *         *         *         *         *

             *         *         *         *         *         *         *         *         *         *
701 ACACTCCAATCGGTGATGGTCCTGTTCTGCTGCCAGACAATCACTATCTGAGCACGCAAAGCGTTCTGTCTAAAGATCCGAACGAGAAACGCGATCATAT 800
    ||||||||||||||||||||||||||||||||||||||||||||||||||||||||||||||||||||||||||||||||||||||||||||||||||||
554 acactccaatcggtgatggtcctgttctgctgccagacaatcactatctgagcacgcaaagcgttctgtctaaagatccgaacgagaaacgcgatcatat 653
          *         *         *         *         *         *         *         *         *         *

             *         *         *         *         *         *         *         *         *         *
801 GGTTCTGCTGGAGTTCGTAACCGCAGCGGGCATCACGCATGGTATGGATGAACTGTACAAATGATGATAATAATCTAGACCAGGCATCAAATAAAACGAA 900
    |||||||||||||||||||||||||||||||||||||||||||||||||||||||||||||
654 ggttctgctggagttcgtaaccgcagcgggcatcacgcatggtatggatgaactgtacaaa~~~~~~~~~~~~~~~~~~~~~~~~~~~~~~~~~~~~~~~ 714
          *         *         *         *         *         *
```

Sanger sequencing results of colony 2, sequence alignment to *sfgfp*. Matches: 713, Mismatches: 1, Gaps: 367, Unattempted: 0.

Supplementary Fig. S6.

```
101 TNCAATAATATGAAAAANNGTAGAGTAANGAGGAGAAATACTNGNNGCGTNAAAGGCGAAGAGCTGTTCACTGGNNTCGTCCCTTATTCTGGTGGAACTG 200
  3 ---------------------------------------------Gcgt-aaaggcgaagagctgttcactggtgtcgtccc-tattctggtggaactg 54

201 GATGGTGATGTCAACGGTCATAAGTTTTCCGTGCGTGGCGAGGGTGAAGGTGACGCAACTAATGGTAAACTGACGCTGAAGTTCATCTGTACTACTGGTA 300
 55 gatggtgatgtcaacggtcataagttttccgtgcgtggcgagggtgaaggtgacgcaactaatggtaaactgacgctgaagttcatctgtactactggta 154

301 AACTGCCGGTACCTTGGCCGACTCTGGTAACGACGCTGACTTATGGTGTTCAGTGCTTTGCTCGTTATCCGGACCATATGAAGCAGCATGACTTCTTCAA 400
155 aactgccggtaccttggccgactctggtaacgacgctgacttatggtgttcagtgctttgctcgttatccggaccatatgaagcagcatgacttcttcaa 254

401 GTCCGCCATGCCGGAAGGCTATGTGCAGGAACGCACGATTTCCTTTAAGGATGACGGCACGTACAAAACGCGTGCGGAAGTGAAATTTGAAGGCGATACC 500
255 gtccgccatgccggaaggctatgtgcaggaacgcacgatttcctttaaggatgacggcacgtacaaaacgcgtgcggaagtgaaatttgaaggcgatacc 354

501 CTGGTAAACCGCATTGAGCTGAAAGGCATTGACTTTAAAGAAGACGGCAATATCCTGGGCCATAAGCTGGAATACAATTTTAACAGCCACAATGTTTACA 600
355 ctggtaaaccgcattgagctgaaaggcattgactttaaagaagacggcaatatcctgggccataagctggaatacaattttaacagccacaatgtttaca 454

601 TCACCGCCGATAAACAAAAAAATGGCATTAAAGCGAATTTTAAAATTCGCCACAACGTGGAGGATGGCAGCGTGCAGCTGGCTGATCACTACCAGCAAAA 700
455 tcaccgccgataaacaaaaaaatggcattaaagcgaattttaaaattcgccacaacgtggaggatggcagcgtgcagctggctgatcactaccagcaaaa 554

701 CACTCCAATCGGTGATGGTCCTGTTCTGCTGCCAGACAATCACTATCTGAGCACGCAAAGCGTTCTGTCTAAAGATCCGAACGAGAAACGCGATCATATG 800
555 cactccaatcggtgatggtcctgttctgctgccagacaatcactatctgagcacgcaaagcgttctgtctaaagatccgaacgagaaacgcgatcatatg 654

801 GTTCTGCTGGAGTTCGTAACCGCAGCGGGCATCACGCATGGTATGGATGAACTGTACAAATGATGATAATAATCTAGACCAGGCATCAAATAAAACGAAA 900
655 gttctgctggagttcgtaaccgcagcgggcatcacgcatggtatggatgaactgtacaaa~~~~~~~~~~~~~~~~~~~~~~~~~~~~~~~~~~~~~~~~ 714
```

Sanger sequencing results of colony 3, sequence alignment to *sfgfp*. Matches: 712, Mismatches: 2, Gaps: 367, Unattempted: 0.

Supplementary Fig. S7.

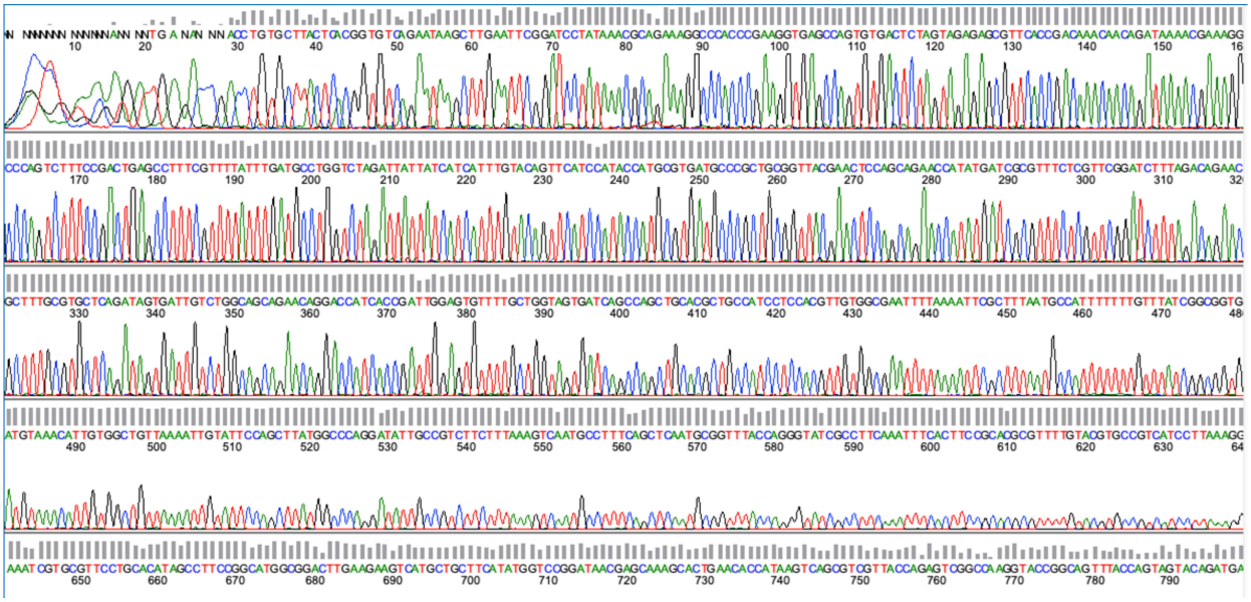

Sequencing chromatograph results of validation of the *gfp* gene.

Supplementary Fig. S8.

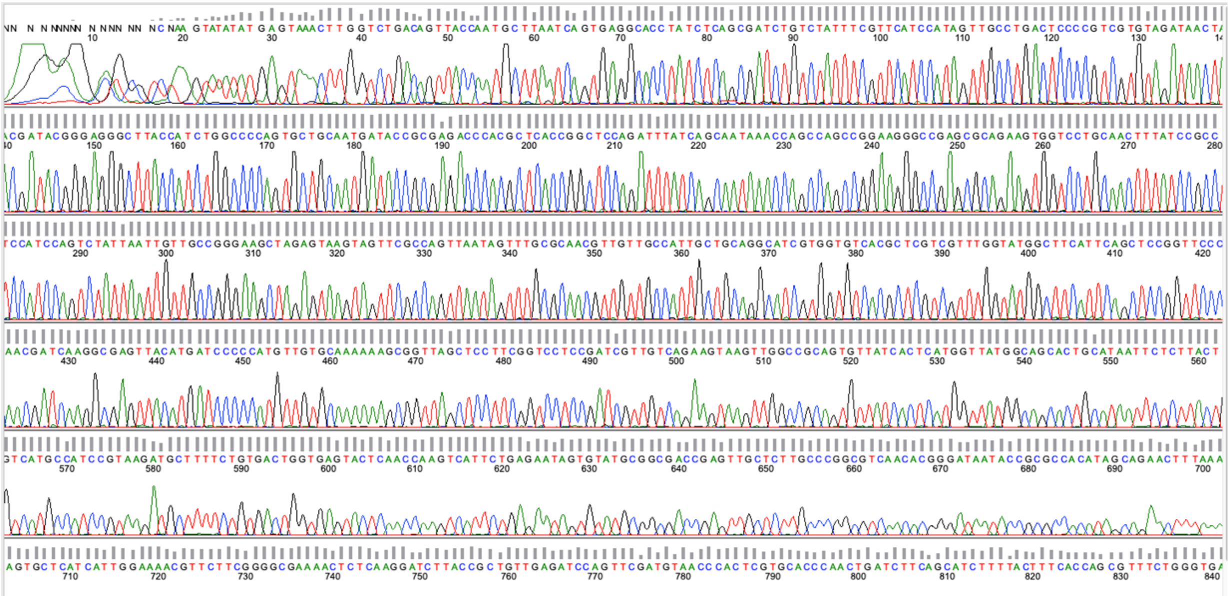

Sequencing chromatograph results of validation of the *amp* gene.

Supplementary Fig. S9.

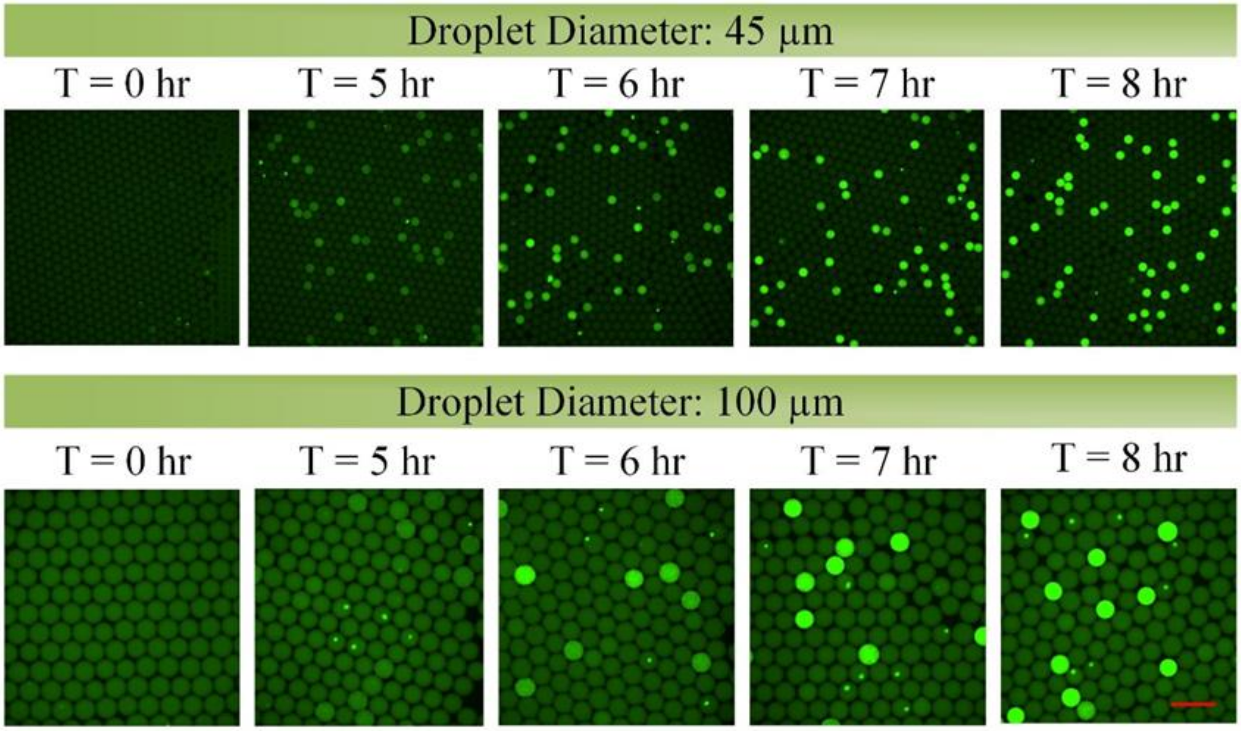

Fluorescence images of bacteria-containing blood after droplet encapsulation with SYBR Green I. Non-fluorescent ampicillin-resistant *E. coli* (CTX-M-14) were spiked into sodium-citrate human blood (10%) mixed with an assay solution (90%) consisting of Terrific Broth (TB) with ampicillin, 10 mM Tris buffer (pH 8.0), 0.05% Saponin, and 1x fluorescence dye (SYBR Green I, BIO-RAD). Initial bacteria concentration was low such that positive droplets contained only one bacterium at T = 0 h. The mixture was encapsulated into microdroplets of 45 μm and 100 μm diameter with a high-throughput droplet generator followed by 8-hour incubation at 37 °C. From the image time sequence, it can be seen that the fluorescence dye SYBR Green penetrated the membrane of the bacteria in droplets binding to the bacteria DNA lighting up bacteria-positive droplets. After 5 h, positive droplets emitted strong fluorescence with thousands of fluorescent bacteria confined within positive droplets. Small fluorescent puncta in droplets are SYBR Green stained nuclei of white blood cells. During 3D droplet sorting, these signals can be rejected by adjusting the width of the detection correlation filter to the droplet size. Scale bar, 200 μm.

Supplementary Fig. S10.

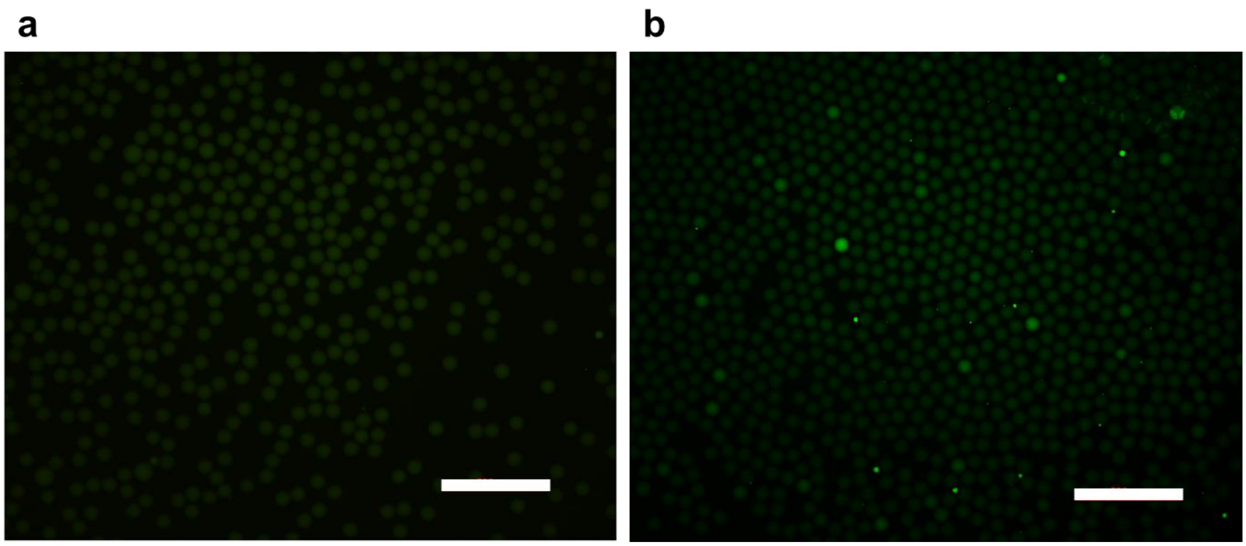

Encapsulation of *E. coli* bacteria with the β-lactamase sensor Fluorocillin. Bacteria were co-encapsulated with Fluorocillin (10 µM) and lysozyme (1 mg/ml) in Luria-Bertani (LB) media and incubated at 37°C for 5-10 min to promote lysis of the bacterial cells by the action of lysozyme. (**a**) Fluorescence image of *E. coli* K12 that did not contain a β-lactamase producing gene. (**b**) Fluorescence image of *E. coli* engineered with ampicillin resistance, a β-lactamase producing gene. Droplet size was approximately 50 µm in diameter. Scale bars, 500 µm.
